# Lysine Acetylation as a Regulatory Mechanism in Cardiometabolic Disease: A Focus on Sirtuins

**DOI:** 10.3390/ph19101557

**Published:** 2026-10-01

**Authors:** Khushali Trivedi, Vernon W. Dolinsky

**Affiliations:** 1 Department of Pharmacology and Therapeutics, University of Manitoba, Winnipeg, MB R3E 0T6, Canada; 2 Diabetes Research Envisioned and Accomplished in Manitoba (DREAM) Research Theme of the Children’s Hospital Research Institute of Manitoba, University of Manitoba, Winnipeg, MB R3E 3P4, Canada

**Keywords:** mitochondria, metabolism, cardiovascular, diabetes, sirtuin

## Abstract

Protein lysine acetylation is a widespread post-translational modification that regulates metabolic enzyme activity and cellular function. Sirtuins (SIRTs) are a family of nicotinamide adenine dinucleotide (NAD^+^)-dependent deacetylases that act as key metabolic sensors coupling cellular energy status to the regulation of protein acetylation. Dysregulation of sirtuin activity, particularly sirtuin 1 (SIRT1) and sirtuin 3 (SIRT3), has been implicated in the pathogenesis of cardiometabolic diseases, including obesity, type 2 diabetes, cardiovascular disease and metabolic dysfunction-associated steatotic liver disease. Under conditions of nutrient excess, alterations in acetyl-coenzyme A (acetyl-CoA) and NAD^+^ availability promote mitochondrial protein hyperacetylation, contributing to impaired mitochondrial respiration. Evidence from genetic and proteomic studies across metabolically active tissues such as the liver, heart, adipose tissue, skeletal muscle and pancreatic β-cells, highlight a central role for sirtuins in maintaining metabolic homeostasis. Therapeutic strategies aimed at improving mitochondrial function through activation of sirtuins have gained significant attention. Early studies focused on natural sirtuin activators, such as resveratrol and honokiol, while subsequent efforts led to the development of synthetic small-molecule activators, although their mechanisms of action remain debated. More recently, small molecule activators of SIRT1, such as SRT2104, and SIRT3, such as 2-acetyl-4-phenylquinoline (2-AQPC) and SZC-6 have been identified. In parallel, NAD^+^ augmenting through supplementation of NAD^+^ precursors such as nicotinamide riboside and nicotinamide mononucleotide has shown similar promising metabolic benefits in preclinical rodent models. Sirtuin-mediated lysine deacetylation represents a central mechanism regulating metabolic function and a promising target for therapeutic intervention in cardiometabolic disease.

## 1. Introduction

Rising rates of obesity are driven by chronic caloric excess and disruptions in energy homeostasis. Obesity and diabetes also significantly increase the risk of cardiovascular disease (CVD), which accounts for nearly 20 million deaths annually [1,2]. Collectively, obesity, diabetes and CVD comprise a spectrum of cardiometabolic disorders that represent the leading cause of mortality worldwide [1,2]. Metabolic dysfunction-associated steatotic liver disease (MASLD), characterized by excessive hepatic lipid accumulation in the presence of cardiometabolic risk factors, is also closely associated with obesity and type 2 diabetes (T2D) and represents an increasingly prevalent manifestation of cardiometabolic disease [3]. Although these disorders have distinct clinical manifestations, they share underlying metabolic abnormalities that arise, in part, from chronic nutrient excess and impaired cellular energy homeostasis [4,5]. These abnormalities include altered substrate utilization, ectopic lipid accumulation, dyslipidemia, insulin resistance, mitochondrial dysfunction and chronic low-grade inflammation [6]. Despite advances in obesity, diabetes and CVD therapeutics, the incidence of cardiometabolic disease continues to rise. Therefore, a better understanding of the cellular mechanisms linking nutrient excess to metabolic and cardiovascular dysfunction is needed to identify new therapeutic targets for cardiometabolic disease.

The aim of this narrative review is to provide a synthesis of the interconnected roles of lysine acetylation, NAD^+^ metabolism and sirtuin activity in the regulation of cellular and mitochondrial metabolism in cardiometabolic disease. Particular emphasis is placed on sirtuin 1 (SIRT1) and sirtuin 3 (SIRT3) across the heart, liver, adipose tissue, skeletal muscle and pancreatic β-cells, with evidence from global and tissue-specific genetic models. Finally, emerging therapeutic strategies targeting sirtuin activity and NAD^+^ metabolism, including pharmacological sirtuin activators and NAD^+^-augmenting approaches, are discussed alongside their current limitations and translational potential.

### 1.1. Mitochondrial Oxidative Metabolism and Dysfunction in Cardiometabolic Disease

Mitochondria are the principal sites of cellular energy metabolism, coordinating the oxidation of carbohydrates, lipids and proteins to generate energy in the form of adenosine triphosphate (ATP) through oxidative phosphorylation (OXPHOS) [7]. These substrates converge on the production of acetyl-coenzyme A (acetyl-CoA) and reducing equivalents, including nicotinamide adenine dinucleotide (NADH) and flavin adenine dinucleotide (FADH_2_), which support the tricarboxylic acid (TCA) cycle and electron transport chain (ETC) [8,9,10]. Efficient mitochondrial oxidative metabolism therefore requires coordinated substrate oxidation and maintenance of the NAD^+^/NADH redox balance.

In cardiometabolic disease, chronic nutrient excess alters mitochondrial substrate metabolism and shifts the NAD^+^/NADH ratio toward a more reduced state [11]. Mitochondrial dysfunction in cardiometabolic disease is characterized by impaired respiration and substrate oxidation, altered mitochondrial dynamics, increased reactive oxygen species (ROS) production and disrupted mitophagy [12,13]. These abnormalities have been observed across multiple metabolically active tissues, including the liver, skeletal muscle, adipose tissue and heart, in both preclinical models and patients with obesity, T2D and CVD [4,5,14]. In the liver, reduced mitochondrial respiration and fatty acid oxidation (FAO) are associated with hepatic steatosis and insulin resistance [15,16,17,18]. In adipose tissue, impaired mitochondrial respiration contributes to dysregulated lipid handling and ultimately ectopic lipid accumulation [19,20]. In pancreatic β-cells, impaired mitochondrial ATP production can disrupt glucose-stimulated insulin secretion (GSIS) [21,22,23]. In the heart, mitochondrial dysfunction contributes to diabetic cardiomyopathy, where increased fatty acid utilization and reduced glucose oxidation contribute to metabolic inflexibility and reduced cardiac efficiency [24,25]. Together, these findings suggest that mitochondrial dysfunction may actively contribute to the development and progression of cardiometabolic disease [4,26].

Changes in nutrient availability can also influence molecular mechanisms that regulate mitochondrial metabolism. Among these, reversible post-translational modifications, particularly lysine acetylation, provide an important link between cellular nutrient status and mitochondrial function.

### 1.2. Lysine Acetylation as a Metabolic Regulatory Mechanism

Lysine acetylation has emerged as an important post-translational modification (PTM) involved in the regulation of oxidative metabolism and mitochondrial function [27,28,29,30].

**Figure 1:**
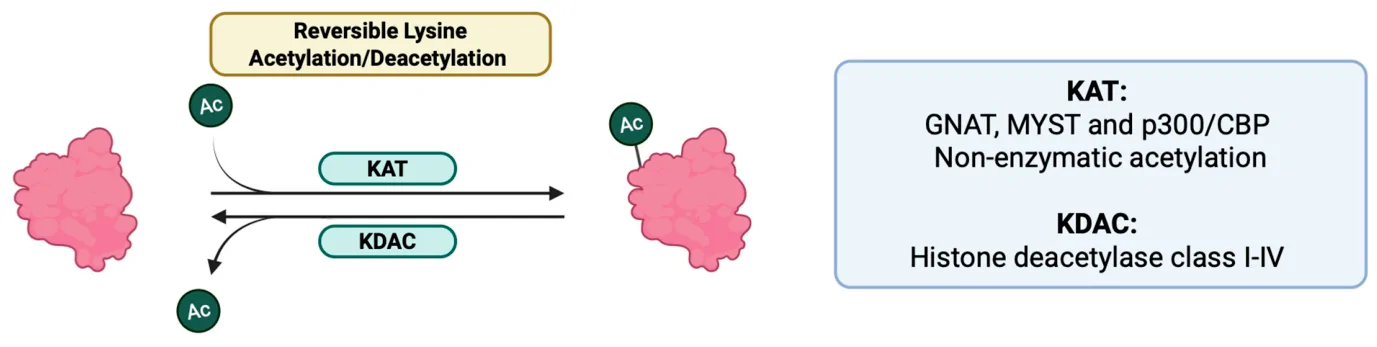
Reversible lysine acetylation and deacetylation. Lysine acetylation is a reversible post-translational modification regulated by the addition and removal of acetyl groups. Lysine acetyltransferases, including GNAT, MYST and p3000/CBP families, catalyze the transfer of acetyl groups to lysine residues, while lysine deacetylases, including histone deacetylase classes I–IV, remove acetyl groups. Ac: acetyl group. KAT: lysine acetyltransferase. KDAC: lysine deacetylase. GNAT: GCN5-related N-acetyltransferase.

An acetyl group (—COCH_3_) is a small two-carbon chemical moiety derived primarily from acetyl-CoA, a central metabolic intermediate, although it can also originate from acetate [31]. In biological systems, acetyl groups are covalently attached to proteins through a reversible post-translational modification known as acetylation, which is catalyzed by lysine acetyltransferase enzymes (KAT) (Figure 1) [32]. Lysine acetylation involves the covalent addition of an acetyl group to the ε-amino group of lysine residues [32]. KATs are commonly classified into three major families based on structural homology and catalytic domain organization: the GCN5-related N-acetyltransferase (GNAT), MYST and p300/CBP families [33]. GNATs are the largest family and regulate transcription and DNA repair [33]. KAT2A/GCN5 catalyzes the transfer of an acetyl group from acetyl-CoA to a lysine residue on histone proteins, reducing chromatin compaction and promoting transcriptional activation [33]. A related protein, GCN5-like protein 1 (GCN5L1) shares structural similarity with GCN5, but primarily localizes to the mitochondria where it can acetylate lysines on mitochondrial proteins [34]. GCN5L1 does not function as a classical KAT as it lacks an acetyl-CoA binding region; however, it facilitates mitochondrial protein acetylation [34,35]. In addition to enzymatic acetylation, non-enzymatic acetylation can occur in environments with high acetyl-CoA concentrations such as the mitochondrial matrix [36]. Because acetyl-CoA levels reflect nutrient availability and increase during caloric excess, protein acetylation is closely linked to cellular metabolic state. Acetylation modifications can neutralize the positive charge on lysine, thereby altering protein structure, stability and/or enzymatic activity [32,37]. In contrast, acetyl groups are removed by a group of enzymes termed deacetylases [32]. Dysregulated protein acetylation has been implicated in metabolic diseases including obesity [38,39], T2D [40] and cardiovascular diseases such as diabetic cardiomyopathy [41], and heart failure [42]. In these conditions, altered lysine acetylation of metabolic and mitochondrial proteins has been associated with impaired substrate metabolism and mitochondrial dysfunction.

## 2. Literature Search

Relevant literature was identified primarily through PubMed using combinations of search terms including “sirtuins,” “SIRT1,” “SIRT3,” “lysine acetylation,” “NAD^+^ metabolism,” “sirtuin activators,” “mitochondrial function,” “obesity,” “type 2 diabetes,” and “cardiovascular disease.” Primary research articles were prioritized when discussing experimental and mechanistic findings, while relevant review articles were used to identify additional primary literature. Where conflicting findings were identified, the original primary studies were examined and differences in experimental model, tissue specificity, disease or dietary conditions, and methodological approach were considered when interpreting the evidence.

## 3. Sirtuins as Central Regulators of Lysine Acetylation and Mitochondrial Function

Lysine deacetylation is mediated by a family of enzymes known as histone deacetylases (HDACs), also broadly referred to as lysine deacetylases (KDACs) (Figure 1) [32]. HDACs catalyze the removal of acetyl groups from lysine residues on target proteins. Historically, deacetylation was studied primarily in the context of chromatin regulation, where removal of acetyl groups from histone tails in the nucleus promotes chromatin compaction and transcriptional regulation [43]. As a result, deacetylases were long considered epigenetic regulators confined largely to the nucleus. However, lysine deacetylation also occurs outside the nucleus [37]. Several non-histone proteins in the cytosol and mitochondria involved in energy metabolism are subject to acetylation modification [27]. In 2006, Kim et al. conducted the first large-scale proteomic survey of lysine acetylation in mouse liver mitochondria [27]. Using nanoscale liquid chromatography-tandem mass spectrometry (HPLC-MS/MS) 277 unique acetylation sites were identified across 133 proteins in mouse liver mitochondria isolated from both fed and fasted animals. Furthermore in 2010, Zhao et al. performed liquid chromatography-tandem mass spectrometry (LC-MS/MS) on human cytosolic and mitochondrial liver fractions and found virtually all enzymes in energy metabolism (glycolysis, TCA cycle, FAO, etc.) are subject to acetylation [28]. The identification of acetylated proteins within mitochondrial matrix demonstrated that lysine acetylation is not restricted to nuclear histones and is a common and widespread PTM within the mitochondria.

Sirtuins are Class III that are structurally and mechanistically distinct from the Class I, II and IV deacetylases [43]. Sirtuins are homologous to the yeast silent information regulator 2 (Sir2) with roles in silencing genes near the telomeres [44,45,46,47]. Interest in sirtuins increased following early studies demonstrating that overexpression of Sir2 extended lifespan by 30% in *Saccharomyces cerevisiae*, highlighting the role of sirtuins in the regulation of longevity and aging [45]. Subsequent studies across multiple organisms further supported a role for sirtuins in lifespan regulation, particularly in the context of calorie restriction (CR) [48]. However, it is now recognized that the biological functions of sirtuins extend far beyond longevity. Rather than relying on zinc for catalytic activity like other HDAC classes, sirtuins require NAD^+^ as an obligate cofactor [49]. In mammals, seven sirtuin isoforms have been identified. Non-mitochondrial sirtuins and their activity are summarized in Table 1.

### 3.1. Overview of the Sirtuin Family

**Figure 2:**
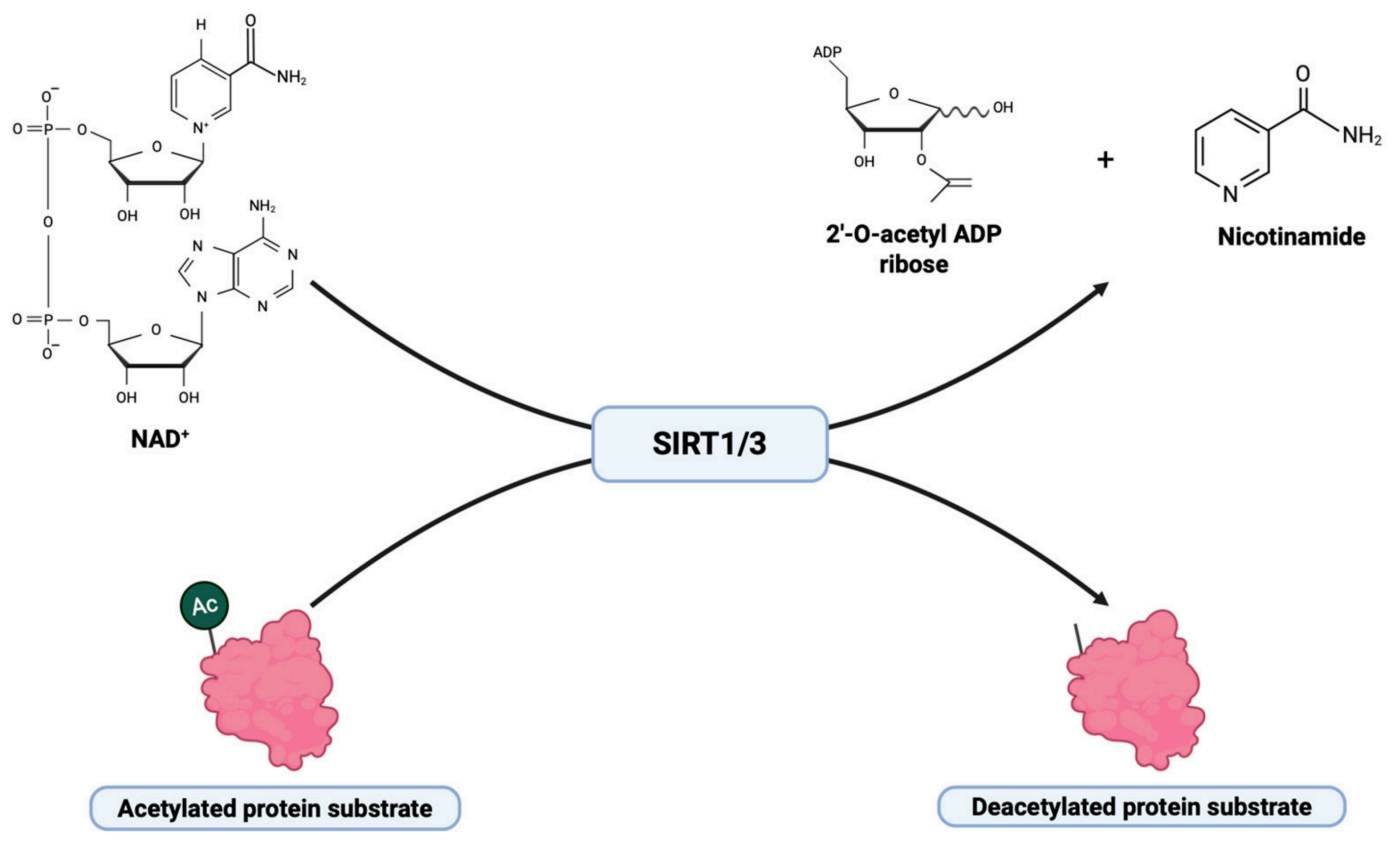
Mechanism of sirtuin-mediated deacetylation. Sirtuins mediate protein deacetylation whereby NAD^+^ is cleaved to generate a deacetylated lysine residue, 2′-O-acetyl-ADP-ribose (OAADRr), and nicotinamide. SIRT1 is located in the cytosol and nucleus and SIRT3 is located in the mitochondria. Ac: acetyl group. SIRT1: sirtuin 1. SIRT3: sirtuin 3. Created in BioRender. Trivedi, K. (2026) https://BioRender.com/8lpea0n.

A defining feature of all sirtuins is their requirement for NAD^+^ as an obligate cofactor [49]. During the deacetylation reaction, NAD^+^ is cleaved to generate a deacetylated lysine residue, 2′-O-acetyl-ADP-ribose (OAADRr) and nicotinamide (Figure 2) [50,51]. Although initially characterized as NAD^+^-dependent deacetylases, sirtuins exhibit diverse catalytic activities beyond deacetylation. Depending on the isoform, these include mono-ADP-ribosyltransferase activity as well as removal of other acyl modifications such as succinyl, malonyl and glutaryl groups [50,51].

There are seven mammalian sirtuin isoforms (SIRT1–7), each with distinct subcellular locations and functional roles. The metabolic function of sirtuins must be considered within the context of substantial tissue-specific differences in mitochondrial abundance and oxidative capacity. Highly oxidative tissues, including cardiac and skeletal muscle, possess substantially different mitochondrial demands and respiratory capacities than tissues with comparatively lower oxidative requirements. The metabolic consequences of altered sirtuin activity are likely influenced not only by sirtuin abundance, but also the underlying mitochondrial landscape of each tissue. Although comparative studies across mammalian species further demonstrate considerable variation in mitochondrial content, mtDNA abundance, and respiratory capacity, a detailed comparative analysis is beyond the scope of this review, and we primarily focus on evidence from rodent studies and transgenic animals.

SIRT1 is the most extensively studied sirtuin and localizes to both the nucleus and cytosol. It exhibits robust NAD^+^-dependent deacetylase activity and regulates several metabolic processes, including glucose and lipid metabolism, fatty acid oxidation and mitochondrial biogenesis [52]. One of the most well-characterized metabolic targets of SIRT1 is peroxisome proliferator-activated receptor gamma coactivator 1-alpha (PGC-1α), which is activated through SIRT1-mediated deacetylation to promote mitochondrial biogenesis and oxidative metabolism [53,54,55].

Sirtuin 2 (SIRT2) is primarily cytosolic and also demonstrates strong NAD^+^-dependent deacetylase activity. It regulates cytoskeletal dynamics through deacetylation of alpha (α)-tubulin [56]. SIRT2 has also been extensively implicated in neuronal function. Sirtuin 6 (SIRT6) is a nuclear sirtuin with both NAD^+^-dependent deacetylase and mono-ADP-ribosyl transferase activities, with substrates including histone H3 lysine 9 acetylation (H3K9Ac) and H3K56Ac [57]. It plays an important role in DNA repair, genomic stability and more recently shown to exhibit deacylase activity towards long chain fatty acyl groups, linking SIRT6 to lipid metabolism [58]. Sirtuin 7 (SIRT7) is nuclear localized, specifically nucleolar and primarily functions as a NAD^+^-dependent deacetylase enzyme in regulating ribosomal DNA transcription and promoting ribosome biosynthesis [59,60]. Its best-characterized substrate is H3K18Ac [59].

**Figure 3:**
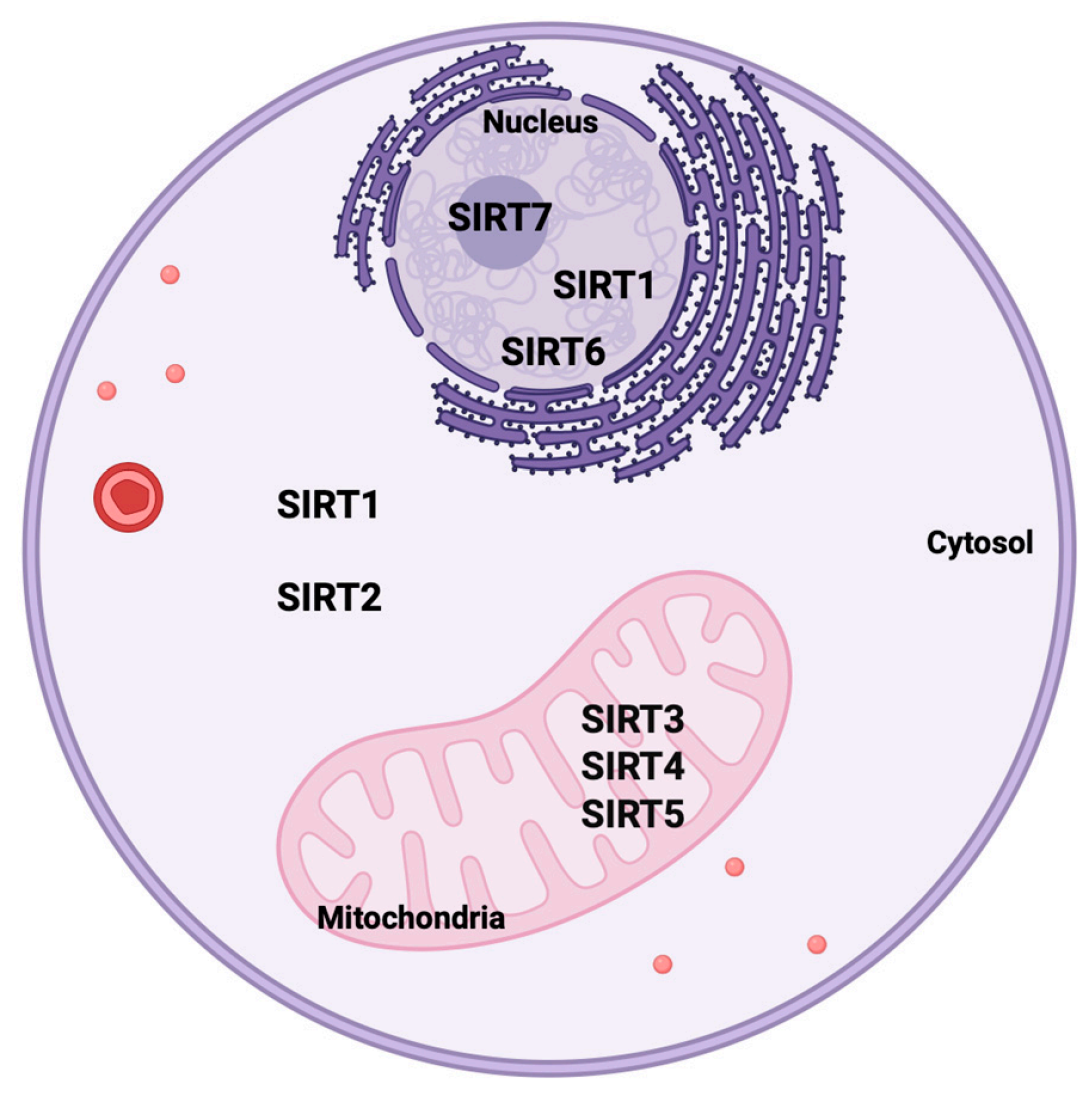
Subcellular location of sirtuin 1–7 localization. SIRT1 and SIRT6 are predominantly localized in the nucleus, SIRT7 is primarily localized in the nucleolus. SIRT1 and SIRT2 are cytosolic, and SIRT3, 4 and 5 are localized to the mitochondrial. SIRT: sirtuin.

In contrast, SIRT3, sirtuin 4 (SIRT4) and sirtuin 5 (SIRT5) localize predominantly to the mitochondria, where they directly regulate mitochondrial protein acetylation (Figure 3) [61].

### 3.2. Mitochondrial Sirtuins

Within the mitochondrial matrix, there are three sirtuins, SIRT3, 4 and 5 (Figure 3) [61]. SIRT4 and SIRT5 exhibit relatively weak lysine deacetylase activity compared to SIRT3. SIRT4 primarily functions as a mono-ADP-ribosyltransferase and lipoamidase, whereas SIRT5 displays robust desuccinylase, demalonylase and deglutarylase activities [62,63]. Acetylome analyses of mitochondrial proteins from mitochondrial sirtuin knockout (KO) models have further clarified their relative roles. Deletion of SIRT3 results in widespread mitochondrial protein hyperacetylation, identifying SIRT3 as the principal mitochondrial deacetylase [64]. These studies also generated both SIRT4 and SIRT5-KO mice, which do not exhibit significant global changes in mitochondrial acetylation, consistent with their limited deacetylase activity. Furthermore, the mitochondrial proteins of SIRT5-KO mice show marked lysine succinylation, confirming the primary role of SIRT5 as a deacylase rather than a classical deacetylase [64,65]. These findings demonstrate that while SIRT4 and SIRT5 regulate specific acyl modifications, SIRT3 is the dominant regulator of mitochondrial lysine acetylation within the mitochondrial acetylome (Table 2).

SIRT3 is widely expressed across tissues but is most abundant in organs with high metabolic demand, such as the liver, brain, skeletal muscle, kidney and heart [66,67]. SIRT3 activity and expression are tightly regulated through multiple mechanisms, including cofactor availability, PTM and transcriptional control. Because SIRT3 requires NAD^+^ as an obligate cofactor, its enzymatic activity is directly influenced by the mitochondrial NAD^+^/NADH ratio [68]. For example, CR elevates mitochondrial NAD^+^ levels and enhances SIRT3 activity [55]. In contrast, nutrient excess, aging and metabolic disease are often associated with reduced NAD^+^ availability, limiting SIRT3 activity [68]. SIRT3 regulates a wide range of mitochondrial enzymes involved in oxidative metabolism, energy production and oxidative stress [27,69,70]. Through deacetylation of key metabolic enzymes, SIRT3 modulates FAO, the TCA cycle, OXPHOS, amino acid metabolism, ketogenesis and mitochondrial antioxidant defense [64]. The functional consequences of SIRT3-mediated deacetylation are determined in part by the upstream mitochondrial protein landscape established through coordinated nuclear and mitochondrial gene expression. Nuclear-encoded respiratory-chain subunits and assembly factors exhibit tissue- and context-dependent expression that contributes to the abundance and assembly of mitochondrial respiratory complexes. Consequently, SIRT3-mediated deacetylation of proteins localized to the mitochondria occurs within a broader regulatory network in which mitochondrial protein expression, complex assembly, and post-translational modification are interconnected.

## 4. Sirtuins in Cardiometabolic Diseases

Given the central role of sirtuins in regulating mitochondrial metabolism, increasing attention has focused on their contribution to cardiometabolic diseases. An early study in yeast demonstrated that the sirtuin homolog Sir2 was required for lifespan extension induced by CR, suggesting that sirtuins function as metabolic sensors linking nutrient availability to cellular adaptation [71]. In parallel, proteomic studies identified extensive lysine acetylation across numerous metabolic enzymes in the liver [27,28]. These findings suggested that lysine acetylation may play a broader role in metabolic regulation and raised the possibility that sirtuins could influence metabolic homeostasis.

Early genetic loss-of-function studies supported this idea [64,72]. Lombard et al. demonstrated that global deletion of SIRT3 resulted in widespread mitochondrial protein hyperacetylation in the liver and brown adipose tissue mitochondria, establishing SIRT3 as a key regulator of the mitochondrial acetylome [64]. Subsequent work by Ahn et al. demonstrated that loss of SIRT3 impaired mitochondrial respiration, with SIRT3-deficient mice exhibiting reduced ATP production in the liver, kidney and heart, linking mitochondrial acetylation to mitochondrial function [72].

Chronic nutrient excess and metabolic stress induced by a high-fat diet (HFD) feeding, increases mitochondrial protein acetylation in several organ systems [38,39,40]. Early studies by Hirschey et al. and Bharathi et al. identified the hyperacetylation of key liver mitochondrial enzymes involved in FAO, which reduces their enzymatic activity, resulting in impaired FAO [39,40]. Similarly, increased mitochondrial protein acetylation has also been observed in the hearts of Wistar rats [41]. An important consideration when interpreting genetic studies of sirtuins is the distinction between whole-body and tissue-specific models. Phenotypes observed following germline deletion may reflect integrated effects across multiple organs and therefore cannot necessarily be attributed to loss of sirtuin activity within an individual tissue. Accordingly, where available, findings from tissue-specific models are considered alongside whole-body models, such as global SIRT1 or SIRT3-KO models, as well as cardiac-specific deletion or overexpression models. Nonetheless, these findings suggest that dysregulation of mitochondrial protein acetylation across various tissues contributes to metabolic dysfunction under nutrient excess.

### 4.1. Cardiovascular Disease

The heart is an energy-demanding organ that predominantly relies on mitochondrial oxidative metabolism to sustain continuous contractile activity [14]. Mitochondrial dysfunction and impaired substrate utilization are recognized as central contributors to the development of cardiac dysfunction [24]. Consequently, sirtuins have emerged as important modulators of cardiovascular physiology.

Increased protein lysine acetylation has been observed in models of diabetic cardiomyopathy and heart failure, suggesting dysregulation of the acetylation-deacetylation balance in the diseased heart [41,73]. Proteomic studies have reported mitochondrial protein hyperacetylation in cardiac tissue of patients with diabetic cardiomyopathy [74,75]. Similar alterations have been extensively studies in rodent models of diabetic cardiomyopathy and heart failure, whereby reduced expression and activity of SIRT1 and/or SIRT3 are also observed [76]. The association between mitochondrial hyperacetylation and reduced sirtuin activity led to the hypothesis that sirtuins are central to the development and progression of CVD.

#### 4.1.1. Role of SIRT1 in Cardiovascular Disease

Among sirtuins, SIRT1 is the most extensively studied in the context of CVD and plays important roles in maintaining metabolic homeostasis and mitochondrial function in the heart.

Experimental models of diabetic cardiomyopathy have demonstrated reduced cardiac SIRT1 expression [77]. Cardiac-specific deletion of SIRT1 resulted in cardiac hypertrophy, impaired cardiac function and insulin resistance, indicating that SIRT1 is required for maintaining normal cardiac metabolism and function under metabolic stress [77]. Furthermore, germ-line deletion of SIRT1 results in ventricular dilation and significantly reduced mitochondrial complex IV activity in the heart, although specific acetylation patterns of mitochondrial proteins were not assessed [78].

Clinical and experimental evidence also supports a role for SIRT1 in regulating cardiac lipid metabolism in diabetes. Reduced SIRT1 expression was observed in the hearts of diabetic patients compared to nondiabetic controls, accompanied by intramyocardial triacylglycerol accumulation [79]. In the same study, the *db*/*db* mouse model for diabetes exhibited decreased cardiac SIRT1 levels, increased myocardial lipid accumulation and impaired cardiac function [79]. Restoration of SIRT1 activity through chronic (four-week) treatment with recombinant SIRT1 improved cardiac function and reduced myocardial triacylglycerol content, highlighting the protective role of SIRT1 in diabetic cardiomyopathy [79].

In an ischemia/reperfusion (I/R) injury mouse model, both SIRT1 protein and gene expression were reduced [80]. Cardiac-specific deletion of SIRT1 results in a larger infarct size after 30 min of ischemia and 24-h reperfusion, whereas cardiac-specific overexpression of SIRT1 is protective against I/R injury [80]. These findings highlight a protective role for SIRT1 in limiting cardiac injury during I/R.

Consistent with these studies, gain-of-function studies have also demonstrated beneficial effects of SIRT1 activation in cardiac cells. Adenoviral overexpression of SIRT1 in neonatal rat cardiomyocytes reduced acetylation of PGC-1α and attenuated hypertrophic markers, including atrial natriuretic factor and α-skeletal actin, following stimulation with phenylephrine [81]. These protective effects were abolished by treatment with a SIRT1 inhibitor, confirming that the antihypertrophic effects were dependent on SIRT1 activity. Similarly, cardiac-specific SIRT1 expression at moderate levels in transgenic mice exhibits cardioprotection characterized by reduced cardiac hypertrophy, fibrosis and dysfunction [82]. However, high levels of SIRT1 expression induces dilated cardiomyopathy with reduced mitochondrial function, indicating that the beneficial effects of SIRT1 are dose-dependent and that excessive SIRT1 activation has off-target effects that are deleterious to the cardiac tissue [82,83].

#### 4.1.2. Role of SIRT3 in Cardiovascular Disease

SIRT3 has been implicated in various cardiovascular diseases such as cardiac hypertrophy, IR injury, myocardial infarction (MI), diabetic cardiomyopathy and heart failure with preserved ejection fraction (HFpEF). Genetic models have provided important insights into the role of SIRT3 in cardiac physiology. Whole-body SIRT3-KO develops cardiac hypertrophy, fibrosis and mitochondrial dysfunction [84,85]. Conversely, cardiac-specific SIRT3 overexpression attenuates cardiac hypertrophy and fibrosis, supporting a cardioprotective role for SIRT3 [85].

Additional studies have demonstrated that loss of SIRT3 exacerbates susceptibility to IR injury. In cardiac-derived H9c2 cells with SIRT3 knockdown, cells were more vulnerable to simulated IR injury and exhibited reduced mitochondrial complex I activity and oxygen consumption, indicating impaired mitochondrial respiration [86]. Similarly, in Langendorff-perfused hearts from whole-body SIRT3-KO mice, functional recovery following IR injury was reduced and infarct size was increased compared to wild-type controls [86]. Increased mitochondrial protein acetylation was accompanied by reduced manganese superoxide dismutase (MnSOD) and complex I in cardiac mitochondria of SIRT3-KO [86]. Following ligation of the left anterior descending coronary artery, SIRT3-KO mice developed more severe cardiac dysfunction compared to controls [87]. In contrast, increased SIRT3 expression improved post-MI cardiac function, suggesting SIRT3 promotes cardiac recovery following ischemic injury [87].

Chen et al. linked SIRT3 to cardiac metabolic remodeling using a pressure-overload model of cardiac hypertrophy induced by transverse aortic constriction (TAC) [88]. In this model, cardiac SIRT3 protein expression was significantly reduced in hypertrophic hearts. Global deletion of SIRT3 produced a phenotype resembling the TAC-induced hypertrophic model characterized by impaired cardiac function, cardiac hypertrophy and increased myocardial lipid accumulation [88]. Mechanistically, SIRT3-KO mice showed reduced FAO and increased acetylation of long-chain acyl-CoA dehydrogenase (LCAD), similar to Hirschey et al. in the liver [38,88]. Conversely, adenoviral overexpression of SIRT3 in cardiomyocytes increased SIRT3 expression and reduced lipid accumulation [88]. Specific acetylation sites on LCAD were not clarified and FAO was not measured in cardiomyocytes following SIRT3 overexpression.

SIRT3 preserved mitochondrial function in models of chemotherapy-induced cardiomyopathy. In H9c2 cardiomyocytes exposed to doxorubicin, reduced SIRT3 expression was associated with increased mitochondrial ROS production. Doxorubicin treatment significantly reduced basal and maximal respiration, whereas restoration of SIRT3 using adenoviral overexpression attenuated oxidative stress and improved mitochondrial respiratory function [89]. These effects were absent in deacetylase-deficient mutant SIRT3 overexpression, suggesting SIRT3 enhances mitochondrial function through its deacetylase activity. More recently, Tomczyk et al. demonstrated that cardiac-specific overexpression of SIRT3 driven by the muscle creatine kinase promoter attenuated doxorubicin-induced dilated cardiomyopathy [90]. Mechanistically, SIRT3 overexpression reduced mitochondrial protein hyperacetylation and H_2_O_2_ production, a mitochondrial ROS, while improving cardiac function [90]. Acetylomic profiling identified widespread changes in mitochondrial metabolic pathways supporting SIRT3 as a global regulator of mitochondrial metabolism and oxidative stress in the failing heart.

Metabolic stress also affects SIRT3 expression and mitochondrial protein acetylation in the heart. C57BL/6 mice on a 60% HFD for 18 weeks exhibit reduced SIRT3 expression, hyperacetylation of FAO enzymes, including LCAD and β-hydroxyacyl-CoA dehydrogenase (β-HAD) and overall increased FAO in cardiac tissue [76]. Whole-body deletion of SIRT3 increased cardiac protein lysine acetylation of FAO enzymes and resulted in greater reliance on FAO [76]. This study also reported that hyperacetylation of FAO enzymes increases their activity. High rates of FAO reduced cardiac efficiency, highlighting the role of cardiac SIRT3 during nutrient excess [91]. Importantly, this study did not perform site-specific mutagenesis of LCAD and β-HAD, making it difficult to determine the direct impact of acetylation on enzyme activity.

In a mouse model of HFpEF, SIRT3 protein expression was reduced and mitochondrial protein hyperacetylation was observed [42]. Cardiac-specific SIRT3-KO mice developed accelerated diastolic dysfunction under HFpEF conditions; however, mitochondrial protein acetylation in SIRT3-KO mice was not assessed [42].

The major findings from experimental studies examining SIRT1 and SIRT3 across different cardiovascular conditions are summarized in Table 3. Despite the predominantly cardioprotective effects reported for SIRT1 and SIRT3, several findings highlight the context-dependent nature of sirtuin activity in the heart. For example, moderate cardiac SIRT1 overexpression attenuates cardiac hypertrophy, fibrosis, and dysfunction, whereas high levels of SIRT1 expression induce dilated cardiomyopathy and mitochondrial dysfunction [82,83], suggesting that the effects of SIRT1 are dependent on the magnitude of expression. Conflicting findings have also been reported regarding SIRT3-mediated regulation of FAO. While several studies demonstrate that increased acetylation of LCAD and β-HAD is associated with reduced enzyme activity and FAO [38,39,88], others report increased activity of these enzymes and greater reliance on FAO under conditions of SIRT3 deficiency and mitochondrial protein acetylation [76]. These discrepancies may reflect differences in experimental models, metabolic conditions and the specific lysine residues examined. Importantly, general measurements of total protein acetylation do not identify the specific lysine residues modified or their acetylation stoichiometry, which may contribute to differences in the reported functional effects of acetylation. Together, these limitations further emphasize that changes in global protein acetylation do not necessarily predict the functional consequences of acetylation at individual sites. Furthermore, many studies examining SIRT1 and SIRT3 rely on whole-body KO models, making it difficult to determine whether the observed cardiac phenotypes arise directly from loss of SIRT1 and/or SIRT3 within cardiomyocytes or indirectly from systemic metabolic alterations.

### 4.2. Metabolic Disease

Increasing evidence suggests that chronic nutrient excess is closely associated with changes in lysine acetylation, particularly within mitochondria [39,40]. Elevated levels of metabolic intermediates such as acetyl-CoA and alterations in the NAD^+^/NADH ratio promote protein hyperacetylation [40]. In the mitochondria, high acetyl-CoA concentrations can drive non-enzymatic acetylation of mitochondrial proteins [36]. At the same time, reduced expression of sirtuins and sirtuin deacetylation activity due to a reduced NAD^+^/NADH ratio have been reported in obesity and T2D [39,40]. Together, increased non-enzymatic acetylation and reduced sirtuin-mediated deacetylation contribute to protein hyperacetylation, which has been associated with impaired mitochondrial function [72]. Experimental studies using loss-of-function and gain-of-function models have demonstrated that several sirtuin isoforms regulate glucose homeostasis, insulin sensitivity and mitochondrial metabolism in metabolic tissues such as the liver, white adipose tissue, skeletal muscle and pancreatic β-cells.

#### 4.2.1. Role of SIRT1 in Metabolic Disease

SIRT1 is highly expressed in metabolically active tissues such as liver, adipose tissue, skeletal muscle and pancreatic β-cells [55,92,93,94].

SIRT1 regulates hepatic glucose and lipid metabolism through deacetylation of transcriptional regulators involved in gluconeogenesis, fatty acid oxidation and lipogenesis [52]. Genetic loss-of-function studies, however, demonstrate that the metabolic effects of hepatic SIRT1 are complex. Knockdown of hepatic SIRT1 using an antisense oligonucleotide (ASO) in a T2D rat model reduced fasting blood glucose levels and hepatic glucose production while improving whole-body insulin sensitivity during a hyperinsulinemic-euglycemic clamp [95]. In contrast, liver-specific deletion of SIRT1 in mice fed a standard chow diet resulted in hyperglycaemia, increased hepatic glucose production, impaired insulin signaling in adipose tissue and skeletal muscle and increased oxidative stress [96]. Conversely, adenoviral overexpression of SIRT1 in the liver improved glucose tolerance in both low-density lipoprotein receptor KO mice fed a high-fat high-sucrose (HFHS) diet and genetically obese (*ob*/*ob*) mice, supporting a protective role for SIRT1 in metabolic dysfunction [97]. Together, these findings demonstrate an important but context-dependent role for hepatic SIRT1 in the regulation of glucose and lipid metabolism.

In white adipose tissue (WAT), SIRT1 regulates lipid metabolism through deacetylation of the nuclear receptor peroxisome proliferator-activated receptor gamma (PPARγ) [98]. Deacetylation of PPARγ represses transcription of downstream genes involved in lipid uptake and storage, thereby promoting lipid mobilization and lipolysis [98]. Adipocyte-specific SIRT1-KO mice fed a HFD for five weeks exhibit hyperacetylation of PPARγ, accompanied by glucose intolerance, hyperinsulinemia and insulin resistance [92]. Interestingly, following 15 weeks of HFD feeding, control mice displayed greater glucose intolerance and insulin resistance, suggesting that the metabolic consequences of adipocyte SIRT1 deficiency may vary with the duration of nutrient excess [92]. Fat-specific SIRT1-KO mice exhibit increased inflammation, whereas global transgenic overexpression of SIRT1 attenuates these responses during HFD feeding [99]. Furthermore, adipose tissue-specific SIRT1 overexpression prevents age-associated declines in insulin sensitivity [100]. Together, these findings support a role for SIRT1 in adipose tissue metabolism; however, adipocyte-specific SIRT1 overexpression has not yet been extensively examined in the context of HFD feeding, leaving the role of SIRT1 in adipose tissue during chronic nutrient excess incompletely understood.

Patients with T2D have reduced mitochondrial respiration and oxidative capacity in skeletal muscle, suggesting mitochondrial dysfunction contributes to the development of insulin resistance [4]. Muscle biopsies from patients with T2D exhibit reduced SIRT1 expression, suggesting that decreased SIRT1 activity may contribute to impaired mitochondrial metabolism in skeletal muscle, although acetylation patterns of proteins were not assessed [101]. Similarly, mice fed a HFD for 16 weeks exhibit impaired glucose tolerance and reduced expression of SIRT1 in gastrocnemius muscle compared to chow-fed controls [102]. Skeletal muscle-specific SIRT1-KO mice exhibit impaired mitochondrial respiration and reduced mitochondrial content [103]. Whereas, overexpression of SIRT1 in skeletal muscle of HFD-fed rats attenuated insulin resistance and increased mitochondrial complex I–IV activities, suggesting improved mitochondrial function [104]. In contrast, other studies reported that muscle-specific overexpression of SIRT1 did not significantly improve glucose tolerance, insulin sensitivity or maximal mitochondrial respiratory capacity in HFD-fed mice [105,106]. Together, these findings suggest that while SIRT1 regulates mitochondrial oxidative metabolism in skeletal muscle, its whole-body metabolic effects appear to be context dependent.

Genetic models support a role for SIRT1 in regulating pancreatic β-cell function. β-cell specific SIRT1-KO mice have demonstrated reduced GSIS, although glucose tolerance remains unchanged [94]. Similarly, pancreatic deletion of SIRT1 using pancreatic duodenal homeobox-1 (Pdx1)-Cre reduces GSIS without inducing overt hyperglycaemia [107]. Conversely, β-cell specific overexpression of SIRT1 enhances GSIS and improves glucose tolerance which has been associated with reduced expression of uncoupling protein 2 (UCP2) and increased mitochondrial ATP production [108]. SIRT1 overexpression also protects β-cells against fatty acid-induced β-cell dysfunction [109]. Together, these findings suggest that SIRT1 support β-cell function and insulin secretion, although the relatively mild metabolic phenotype following SIRT1 deletion suggests the presence of compensatory mechanisms.

Moderate global overexpression of SIRT1 in mice fed a low-fat diet for 20 weeks does not improve glucose tolerance; however, when these mice are challenged with a HFD, glucose tolerance is improved [110]. This finding is also supported by additional studies showing that global SIRT1 overexpression in HFD-fed mice enhances insulin sensitivity and reduced hepatic glucose output compared to non-transgenic mice, demonstrating a protective role against metabolic stress [111]. Similarly, SIRT1 transgenic mice fed a HFD showed improved glucose tolerance, reduced inflammation, and protected against hepatic steatosis [112]. Notably, in many of these global and tissue-specific KO and overexpression models, acetylation or deacetylation of known SIRT1 targets were not directly assessed, making it difficult to determine whether the observed phenotypes are mediated through canonical SIRT1 deacetylase activity. Overall, these findings suggest, global SIRT1 overexpression provides greater protection from HFD-induced obesity and metabolic dysfunction than tissue specific overexpression, indicating that systemic metabolic regulation by SIRT1 across multiple metabolic tissues is likely required to see the full benefit.

#### 4.2.2. Role of SIRT3 in Metabolic Disease

SIRT3 is highly expressed in metabolically active organs such as the brain, liver, heart, muscle and brown adipose tissue [66]. Dysregulation of mitochondrial protein acetylation has been reported in obesity and T2D with SIRT3 being the primary deacetylase in the mitochondria, altered SIRT3 activity may contribute to metabolic dysfunction [40,113,114]. Using isolated liver mitochondria and label-free quantitative mass spectrometry-based acetyl-proteomics, Rardin et al. revealed a substantial increase in lysine acetylation across numerous mitochondrial proteins in the absence of SIRT3 [115]. These changes were not restricted to a single pathway but rather spanned multiple metabolic processes, including FAO, the TCA cycle and OXPHOS [115]. Consistent with these findings, SIRT3 deletion leads to reduced mitochondrial oxidative capacity, decreased respiration and increased ROS across metabolic tissues including the liver, skeletal muscle and adipose tissue [38,113,116,117,118]. Notably, SIRT3-KO mice on a chow diet do not display overt metabolic dysfunction [119].

Liver: The earliest studies characterizing the mitochondrial acetylome were performed in liver tissue or isolated liver mitochondria, where large-scale proteomic analyses revealed extensive lysine acetylation across numerous metabolic enzymes [27,28,38,40,67,115]. Interest in the role of SIRT3 in metabolic disease increased when studies reported that hepatic SIRT3 protein expression was reduced in rodents fed a HFD [113]. Knockdown of SIRT3 in HepG2 hepatocytes using small interfering RNA (siRNA) reduced oxygen consumption rates, indicating impaired mitochondrial respiration [113]. HFD feeding also reduced the expression of SIRT3 and PGC-1α, which regulates SIRT3 expression [120].

A HFD causes significant hepatic mitochondrial protein hyperacetylation in mice, which is further exacerbated in whole-body SIRT3-KO mice [40,72,114]. Whole body SIRT3-KO mice also develop accelerated obesity, insulin resistance, hepatic steatosis, reduced ATP and mitochondrial respiration [40,72,114]. These findings suggest that reduced hepatic SIRT3 expression during nutrient excess may contribute to mitochondrial dysfunction and metabolic disease, prompting the development of liver-specific SIRT3-KO models. Surprisingly, liver-specific SIRT3-KO mice exhibit marked hyperacetylation of mitochondrial metabolic proteins but do not display profound metabolic abnormalities, suggesting the deletion of SIRT3 in the liver on its own is not sufficient to contribute to glucose intolerance and steatosis [121]. In ex vivo studies using primary hepatocytes, overexpression of SIRT3 significantly reduced accumulation of triglycerides and increased mitochondrial respiratory capacity [122]. However, in vivo hepatic overexpression of SIRT3 in mice fed a HFD provided partial improvements in whole-body glucose tolerance or tissue triglyceride accumulation [122]. These findings suggest that hepatic SIRT3 alone may not be sufficient to confer protection against systemic metabolic stress.

In humans, SIRT3 expression was downregulated in the subcutaneous adipose tissue of obese twins [123]. Furthermore, SIRT3 protein expression was significantly reduced in visceral adipose tissue of diabetic *db*/*db* mice [124]. Overexpression of SIRT3 in adipocytes using plasmid vectors increased ATP production through increased mitochondrial fusion [125]. However, adipocyte-specific SIRT3-KO mice exhibit normal mitochondrial function, glucose tolerance and insulin sensitivity when maintained on either a chow diet or a HFD for up to 18 months [124]. These findings suggest that adipose tissue SIRT3 deficiency alone is unlikely to account for the metabolic phenotype observed in germline SIRT3-KO mice.

Reduced SIRT3 expression has also been reported in skeletal muscle in metabolic disease. Skeletal muscle from obese pregnant women exhibits reduced SIRT3 expression and activity, accompanied by hyperacetylation of the SIRT3 target and antioxidant enzyme MnSOD [126]. In rodent models, HFD feeding (42% fat) for 24 weeks significantly reduces SIRT3 gene and protein expression in skeletal muscle [118]. Knockdown of SIRT3 in C2C12 myoblasts using siRNA reduces mitochondrial respiratory capacity, increases intracellular ROS and impairs insulin signaling [117,118]. Germline SIRT3-KO mice display hyperacetylation of mitochondrial proteins in skeletal muscle, which is further exacerbated following 12 weeks of HFD feeding (60% fat) [119]. This is accompanied by reduced mitochondrial respiration in the muscle [119]. However, despite these impairments, insulin signaling in the muscle remains largely normal, suggesting that the mechanisms underlying insulin resistance are not fully understood in SIRT3-KO mice. Skeletal muscle-specific SIRT3-KO mice exhibit significant mitochondrial protein hyperacetylation, but do not develop major metabolic dysfunction such as glucose intolerance and insulin resistance similar to the liver-specific SIRT3-KO model [121]. Similarly, muscle-specific overexpression of SIRT3 in rats using adeno-associated virus was not sufficient to improve insulin sensitivity of chow- or four-week HFD-fed rats, as assessed by hyperinsulinemic-euglycemic clamp studies [127]. These findings suggest that deficiency of skeletal muscle SIRT3 alone is not sufficient to explain the metabolic phenotype observed in whole-body SIRT3-KO mice.

SIRT3 has also been implicated in pancreatic β-cells. In islets isolated from T2D patients, *SIRT3* mRNA expression is significantly reduced [128]. Exposure of INS-1 cells to palmitate reduces SIRT3 expression and significantly impairs insulin secretion, whereas restoration of SIRT3 expression reverses these effects [129]. Knockdown of SIRT3 in INS-1 β-cell lines using siRNA suppresses insulin secretion [128]. On the other hand, complete deletion of SIRT3 using CRISPR/Cas9 in INS-1 cells leads to hyperacetylation of mitochondrial proteins without affecting glucose and pyruvate-stimulated insulin secretion [130]. The precise role of SIRT3 in β-cells is complex and has not been examined in detail in animal model systems.

Studies examining SIRT3 overexpression have produced mixed results. Mice with enterocyte-specific overexpression of SIRT3 exhibit improved glucose tolerance following 10 weeks of HFD feeding, suggesting a potential role for intestinal SIRT3 in metabolic regulation [131]. In contrast, systemic overexpression of SIRT3 using adeno-associated viral vectors does not significantly alter body weight or blood glucose levels in mice fed a HFD for six weeks and treated with STZ to induce diabetes, indicating limited metabolic benefit under these conditions [132]. Similarly, adenoviral overexpression of SIRT3 in genetically diabetic *db*/*db* mice does not significantly affect body weight or blood glucose levels [133]. Collectively, these findings indicate that the metabolic role of SIRT3 in obesity and T2D merits further study.

Representative findings from global and tissue-specific genetic models examining the effects of sirtuins on glucose homeostasis, insulin sensitivity and mitochondrial metabolism are summarized in Table 4. Despite substantial evidence supporting protective metabolic roles for SIRT1 and SIRT3, several conflicting findings highlight the context-dependent nature of their effects. For SIRT1, hepatic loss-of-function studies have produced opposing effects on glucose homeostasis, with hepatic SIRT1 knockdown improving insulin sensitivity and reducing hepatic glucose production in a T2D rat model [95], whereas liver-specific SIRT1 deletion in mice resulted in hyperglycaemia and increased hepatic glucose production [96]. Similarly, the metabolic consequences of adipocyte SIRT1 deficiency vary with the duration of HFD feeding [92], while skeletal muscle SIRT1 overexpression has produced inconsistent effects on glucose homeostasis and mitochondrial function [104]. Discrepancies are particularly evident for SIRT3, as whole-body SIRT3 deletion is associated with obesity, insulin resistance, hepatic steatosis, and mitochondrial dysfunction [40,72,114], whereas liver-, adipocyte- and skeletal muscle-specific SIRT3 deletion results in mitochondrial protein hyperacetylation with comparatively mild or absent systemic metabolic abnormalities [121]. These findings suggest that increased lysine acetylation alone may be insufficient to drive cardiometabolic disease and that its functional consequences are likely dependent on the specific proteins and lysine residues affected, the tissue in which these modifications occur and the broader metabolic context. Thus, rather than representing a uniform driver of metabolic dysfunction, altered lysine acetylation may contribute to disease in a context- and site-specific manner.

Conflicting findings have also been reported in pancreatic β-cells, where SIRT3 knockdown impairs insulin secretion and complete SIRT3 deletion increases mitochondrial protein acetylation without altering glucose-stimulated insulin secretion [128,130]. These differences may reflect variation in the extent and duration of sirtuin manipulation, compensatory responses to chronic genetic deletion, tissue-specific functions, dietary composition and duration, disease severity, and differences between experimental models. Furthermore, systemic phenotypes observed in whole-body models likely reflect coordinated effects across multiple metabolic tissues that may not be reproduced by manipulating SIRT1 or SIRT3 within a single tissue. Together, these findings emphasize that the metabolic consequences of SIRT1 and SIRT3 modulation are highly dependent on tissue and highlight the importance of integrating tissue-specific and systemic approaches when defining their roles in obesity and T2D.

### 4.3. Cardiometabolic Disease and Reduced NAD^+^

Sirtuins use NAD^+^ as an obligate cofactor for their catalytic activity, linking their enzymatic function directly to the cells metabolic state [49]. Consequently, changes in NAD^+^/NADH ratio influence sirtuin activity [68]. Under conditions of nutrient excess, increased flux through glycolysis and the TCA cycle elevates NADH levels, thereby lowering the NAD^+^/NADH ratio and reducing sirtuin activity [68]. Declining NAD^+^ levels have long been studied in the context of aging and longevity, where reductions in NAD^+^ availability were associated with decreased sirtuin activity [134]. Notably, CR increases NAD^+^ levels and enhances sirtuin activity which contributes to beneficial metabolic effects [53]. However, emerging evidence indicates that NAD^+^ depletion is not limited to aging and may also occur in cardiometabolic diseases.

Several studies discussed earlier have reported reduced NAD^+^ levels in cardiometabolic diseases. Early work demonstrated decreased NAD^+^ levels in tissues exposed to chronic hyperglycemia, linking altered redox balance to diabetes [135]. In mouse models of diet-induced obesity and genetic models of obesity, NAD^+^ levels are significantly reduced in addition to sirtuin activity and increased protein acetylation in several tissues [114,136,137,138,139]. These findings support the concept that reduced NAD^+^ availability contributes to impaired sirtuin activity under conditions of nutrient excess. Consistent with this idea, reduced expression of enzymes involves in NAD^+^ biosynthesis is also reduced in obesity, further limiting NAD^+^ availability [123].

## 5. NAD^+^ Metabolism

Cellular NAD^+^ levels are dynamically regulated and responds to changes in nutrient availability and metabolic demand. Because NAD^+^ serves as a cofactor for numerous metabolic enzymes, changes in NAD^+^ availability influences metabolic pathways as well as protein acetylation [49]. NAD^+^ directly affects the activity of NAD^+^ dependent enzymes, including sirtuins linking cellular redox to metabolic regulation [49]. To maintain adequate NAD^+^ levels, cells must rely on several biosynthesis pathways.

### 5.1. NAD^+^ Biosynthetic Pathways

**Figure 4:**
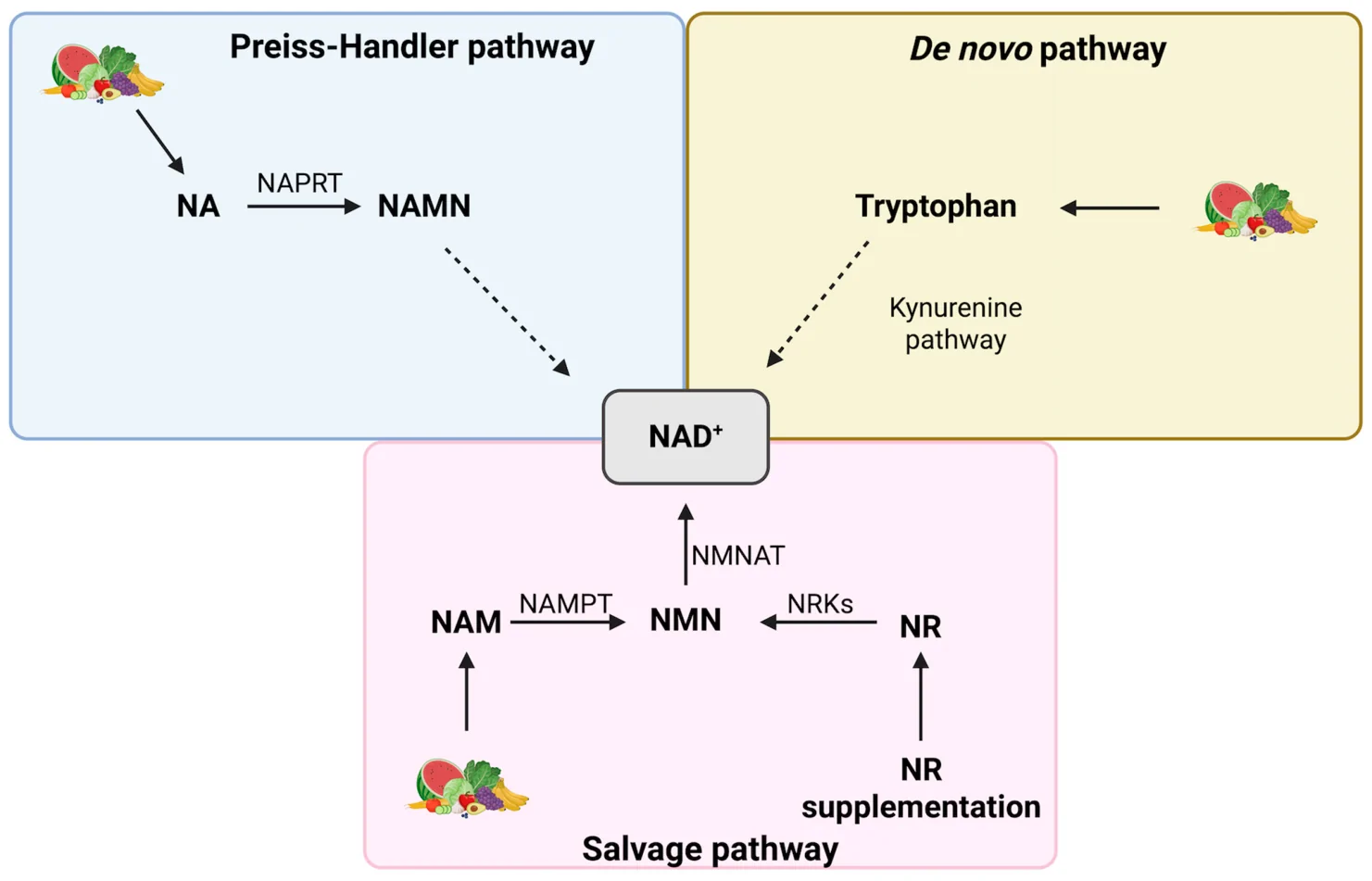
NAD^+^ biosynthetic pathways. NAD^+^ can be synthesized through the de novo, Preiss-Handler and salvage pathways. Solid arrows indicate enzymatic conversation steps, while dashed arrows indicate multiple intermediate reactions that are not shown. NA: nicotinic acid. NAD^+^: nicotinamide adenine dinucleotide (oxidized). NAM: nicotinamide. NAMN: nicotinic acid mononucleotide. NAMPT: nicotinamide phosphoribosyltransferase. NAPRT: nicotinate phosphoribosyltransferase. NMN: nicotinamide mononucleotide. NMNAT: nicotinamide mononucleotide adenylytransferases. NR: nicotinamide riboside. NRKs: nicotinamide riboside kinases.

In mammalian cells, NAD^+^ can be generated through three major independent biosynthetic pathways that use different precursor molecules (Figure 4) [68,140]. The *de novo* pathway (or kynurenine pathway) synthesizes NAD^+^ in the liver from dietary amino acid *L*-tryptophan to generate NAD^+^ through a series of steps [140]. The Preiss-Handler pathway generates NAD^+^ from dietary nicotinic acid (niacin), which is converted to nicotinic acid mononucleotide (NAMN) by nicotinate phosphoribosyltransferase (NAPRT) and through a series of steps to NAD^+^ [68,140]. While these pathways contribute to NAD^+^ production, they play a smaller role in maintaining NAD^+^ pools in the cell. The predominant mechanism for maintaining intracellular NAD^+^ levels is through the nicotinamide salvage pathway, which recycles nicotinamide (NAM), also found from dietary sources and generated during NAD^+^-consuming reactions (sirtuin deacetylase reactions) [68,140]. In this pathway, NAM is converted to nicotinamide mononucleotide (NMN) by the rate-limiting enzyme nicotinamide phosphoribosyltransferase (NAMPT), which is then converted to NAD^+^ by nicotinamide mononucleotide adenylytransferases (NMNATs) [68,140]. Female mice with NAMPT-KO have reduced NMN in their plasma and exhibit glucose intolerance and impaired GSIS [141]. Due to the deacetylase activity of sirtuins, NAM is produced as a by-product. Therefore, the salvage pathway plays a central role in maintaining NAD^+^ pools.

### 5.2. NAD^+^ Consumption

In addition to biosynthesis, cellular levels of NAD^+^ are regulated through its consumption by NAD^+^-dependent enzymes. These enzymes include sirtuins, poly(ADP-ribose) polymerases (PARPs) involved in DNA repair, and NAD^+^ glycohydrolases, such as CD38 and CD157 involved in calcium signaling [68,140]. Increased activity of NAD^+^ consumers can significantly deplete intracellular NAD^+^ pools; thus, a balance between NAD^+^ synthesis and consumption is required to maintain metabolic homeostasis.

### 5.3. Subcellular NAD^+^ Pools

Within the cell, NAD^+^ is compartmentalized in the nucleus, cytosol and mitochondria [140,142]. While NAD^+^ can be readily exchanged between the nucleus and cytosol, the inner mitochondrial membrane restricts exchange with the mitochondrial NAD^+^ pool [142]. Mitochondrial NAD^+^ availability is maintained through both intramitochondrial metabolism and transport of NAD^+^ into the mitochondrial matrix by SLC25A51 [143]. This compartmentalization is particularly relevant to sirtuins, which are also localized to distinct cellular compartments. Changes in NAD^+^ availability within specific cellular compartments can differentially alter the activity of individual sirtuin isoforms.

## 6. Therapeutic Targeting of Sirtuins and NAD^+^ Metabolism

**Figure 5:**
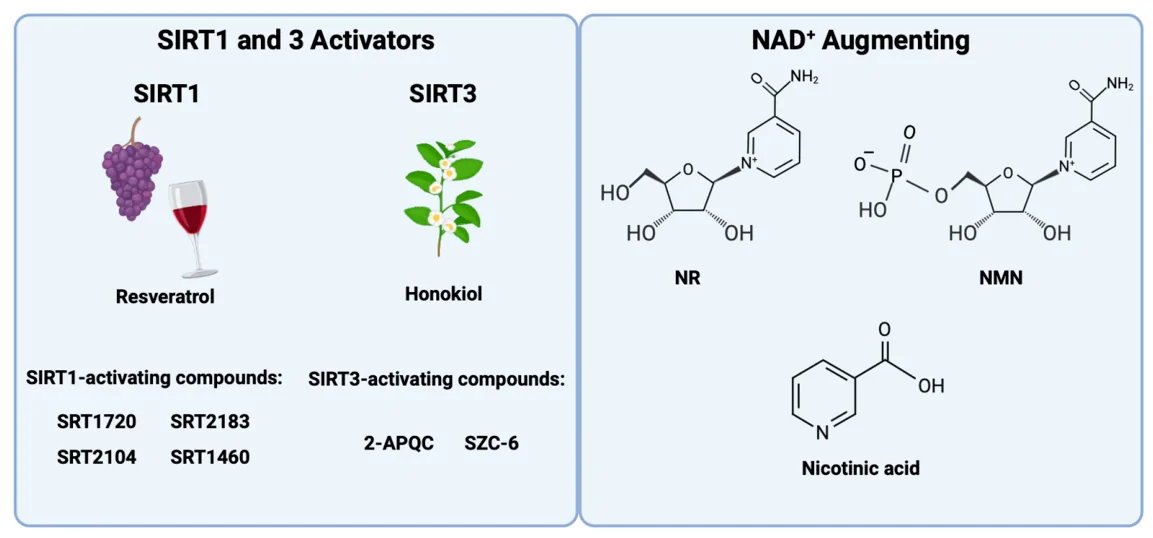
Pharmacological modulators of SIRT1 and SIRT3 and NAD^+^-augmenting precursors. Resveratrol and the synthetic SIRT1-activating compounds SRT1720, SRT2183, SRT2104, and SRT1460 are shown as pharmacological modulators of SIRT1. Honokiol and the SIRT3-activating compounds 2-APQC and SZC-6 are shown as pharmacological modulators of SIRT3. SRT1720, 2104, 2183, 1460: synthetic SIRT1-activating compounds. NAD^+^: nicotinamide adenine dinucleotide (oxidized). NMN: nicotinamide mononucleotide. NR: nicotinamide riboside. SIRT1: sirtuin 1. SIRT3: sirtuin 3. 2-APQC: 2-acetyl-4-phenylquinoline derivative.

Given the central role of sirtuins in regulating mitochondrial function and metabolic homeostasis, NAD^+^ metabolism has emerged as potential therapeutic targets for cardiometabolic diseases. These findings have led to increasing interest in strategies aimed at restoring sirtuin activity and/or enhancing intracellular NAD^+^ levels. Therapeutic approaches targeting sirtuins include compounds that directly enhance sirtuin catalytic activity, as well as agents that increase sirtuin expression or indirectly enhance sirtuin activity through modulation of cellular signaling pathways. Additional therapeutic strategies include augmenting NAD^+^ levels through supplementation with NAD^+^ precursors (Figure 5).

### 6.1. Naturally Occurring Sirtuin Modulators

Based on the observation that SIRT1 mediates metabolic adaptations associated with CR and longevity in yeast, early therapeutic strategies targeting sirtuins focused on identifying compounds capable of enhancing sirtuin activity [144]. One of the first molecules reported to activate SIRT1 was resveratrol (RESV), a naturally occurring polyphenol found in grapes and red wine [144,145]. RESV was initially proposed to enhance SIRT1 activity by lowering the Km for its substrate [144]. Early studies demonstrated that RESV increased SIRT1 activity in vitro and produced metabolic effects in vivo resembling CR, including improved mitochondrial function, increased mitochondrial copy number and exerted protection against diet-induced obesity and insulin resistance in rodent models [146,147,148,149]. These findings generated significant interest in RESV and SIRT1 as a potential therapeutic agent for cardiometabolic diseases. However, the mechanism by which RESV activates SIRT1 has been the subject of considerable controversy. Early studies demonstrating SIRT1 activation relied on in vitro assays using substrates containing a fluorescent moiety (Fluor de Lys) [150]. RESV enhanced SIRT1-mediated deacetylation only in the presence of these fluorescent substrates, with no effect observed on native substrates lacking the fluorophore, suggesting that the apparent activation was an assay-dependent artifact rather than a true physiological effect [150]. Subsequent studies further questioned whether RESV directly activates SIRT1 under physiological conditions [149,151,152].

Accumulating evidence suggests that many of the metabolic effects of RESV occur indirectly through modulation of cellular energy-sensing pathways, particularly the SIRT1-liver kinase B-1 (LKB1)-AMP-activated protein kinase (AMPK) axis. AMPK is activated under conditions of low cellular energy, such as increased AMP/ATP ratio [153]. RESV has been shown to improve glucose tolerance, insulin sensitivity and mitochondrial biogenesis; however, these effects are abolished in AMPKα1 deficient mice, indicating that AMPK is required for RESV-mediated metabolic benefits [149]. RESV can activate AMPK through multiple pathways, including the mild inhibition of Complex III of the ETC [146,154,155].

There is also significant crosstalk between SIRT1 and AMPK signaling. SIRT1-mediated deacetylation of LKB1 promotes its activation, thereby enhancing AMPK signaling [156,157]. Furthermore, AMPK can increase intracellular NAD^+^ levels, indirectly activating SIRT1 [154]. Downstream, both SIRT1 and AMPK converge on common targets such as PGC-1α, with AMPK activating PGC-1α through phosphorylation and SIRT1 through deacetylation, highlighting their complementary roles as energy sensors [154]. Furthermore, David Sinclair’s group later demonstrated that SIRT1 is required for AMPK activation at moderate doses of RESV, whereas at higher doses, RESV activates AMPK independently of SIRT1 [54]. These findings support the growing consensus that RESV functions as a metabolic modulator, rather than a direct and specific activator of SIRT1.

In addition to RESV, other natural compounds have been identified that modulate mitochondrial sirtuin activity and signaling. Quercetin, a plant flavonoid activated human SIRT1 in vitro [144,158]. Treatment of rat insulinoma cells, INS-1E with quercetin improves GSIS and protects β-cell function through reduced oxidative stress [159,160]. Preclinical studies showing 10 mg/kg/day oral administration of quercetin for six weeks decreased blood glucose in STZ-induced diabetic Wistar rats [160]. Honokiol, a biphenolic compound derived from the bark of *Magnolia* species and used in traditional Asian medicine, has been reported to enhance SIRT3 signaling and improve mitochondrial function [161,162]. Honokiol treatment enhances mitochondrial respiration, reduces oxidative stress and protects against cardiac and metabolic dysfunction in rodent models [41,162]. These effects are associated with increased SIRT3 expression in cardiac tissue and deacetylation of the mitochondrial SIRT3 target, MnSOD, resulting in enhanced antioxidant activity [162]. However, whether honokiol directly activates SIRT3 catalytic activity remains unclear.

Despite the promising metabolic effects of these naturally occurring compounds, several limitations remain. These polyphenols exhibit relatively low bioavailability due to interaction with other foods and phase I and II liver metabolism [163]. Additionally, these compounds exert pleiotropic benefits such as anti-oxidant, anti-inflammatory and anti-aging through multiple signaling pathways beyond SIRT1 and AMPK, making it difficult to determine whether their beneficial effects are mediated through direct sirtuin activation [164]. Collectively, these limitations highlight the need for the development of more potent and selective compounds that directly target sirtuin activity.

### 6.2. Synthetic Small-Molecule Sirtuin Activators

In response to the growing controversy of whether RESV and other polyphenols directly activate SIRT1, several groups developed synthetic SIRT1-activating compounds (STACs) designed to more selectively enhance SIRT1 activity [144,148]. Early work identified several candidate compounds through high-throughput screening approaches, which were reported to enhance SIRT1 activity and improve metabolic function in preclinical models [144,148]. These compounds, including SRT1720, SRT2183, SRT1460 and SRT2104, are structurally unrelated to RESV and demonstrated greater potency than RESV in initial studies with improvements in mitochondrial function and protection against HFD-induced obesity and lifespan extension in rodent models [165,166,167,168]. In a phase II, randomized, placebo-controlled, double-blind, multi-dose study using SIRT1 activator SRT2104 (0.25, 0.5, 1.0, 2.0 g/day for 28 days) in patients with T2D, SRT2104 was well tolerated, but was not dose-proportional and did not improve glucose or insulin control [169]. In another small, randomized, double-blind, placebo-controlled study, once-daily oral administration of SRT2104 (2.0 g/day) improved arterial stiffness in otherwise healthy cigarette smokers but showed no effects on blood glucose [170]. Despite promising preclinical findings, translation to clinical studies has been limited.

The mechanism of action of STACs has remained controversial. Pacholec et al. demonstrated that SRT1720, SRT2183 and SRT1460 did not directly activate SIRT1 when assessed using assays with native substrates but instead exhibited activation only in the presence of fluorescently labeled substrates, similar to earlier findings with RESV [151]. Subsequent studies showed that STACs can indeed activate SIRT1 using substrates lacking the fluorescent tag; however, bulky hydrophobic amino acids were used instead and STAC-mediated activation of SIRT1 was again challenged [171,172]. These results raised similar concerns regarding the ability of STACs to directly activate SIRT1 under physiological conditions.

Subsequent studies have suggested that some STACs, particularly SRT1720, may mediate their metabolic benefits through mechanisms similar to RESV, including activation of AMPK, rather than exclusively through direct binding and activation of SIRT1 [173]. The extent to which synthetic STACs function as true direct activators of SIRT1 currently remains an area of ongoing debate.

In addition to targeting SIRT1, efforts have also been made to develop small-molecule activators of mitochondrial sirtuins, particularly SIRT3. In contrast to natural compounds such as honokiol, that increase SIRT3 expression, synthetic compounds such as 2-acetyl-4-phenylquinoline derivative (2-APQC) and SZC-6 directly interact with SIRT3 and enhance its deacetylase activity [174,175]. 2-APQC binds to an allosteric site (Pocket L) near the NAD^+^-binding domain of SIRT3, thereby enhancing its deacetylase activity and improving mitochondrial function [174]. Similarly, SZC-6 binds within an allosteric pocket located in the cleft between the two domains of SIRT3, the Rossmann Fold domain where NAD^+^ binds and smaller helical domain involved in positioning the acetylated lysine substrate [175]. Both compounds have demonstrated improvements in mitochondrial function in preclinical models; however, in vivo efficacy, specificity and long-term safety require further investigation. The major findings, therapeutic limitations and current stage of development of SIRT1- and SIRT3-activating compounds are summarized in Table 5.

### 6.3. NAD^+^ Augmenting

Chronic exposure to nutrient excess is associated with reduced NAD^+^ levels in multiple metabolically active tissues, including the liver, heart, skeletal muscle and adipose tissue [138,176]. Increasing intracellular NAD^+^ levels emerged as a therapeutic strategy to improve metabolic function by enhancing sirtuin activity since sirtuins require NAD^+^ as an obligate cofactor [49]. Early studies demonstrated that the direct administration of NAD^+^ can influence metabolic and transcriptional programs, consistent with the role of NAD^+^ as a metabolite that oscillates across the day-night cycle [177,178]. However, the extent to which exogenously administered NAD^+^ can efficiently enter the cells remains unclear due to its size and charged nature, raising the possibility that its effects may be mediated through extracellular metabolism. These limitations led to the development of alternate NAD^+^ boosting strategies.

**Figure 6:**
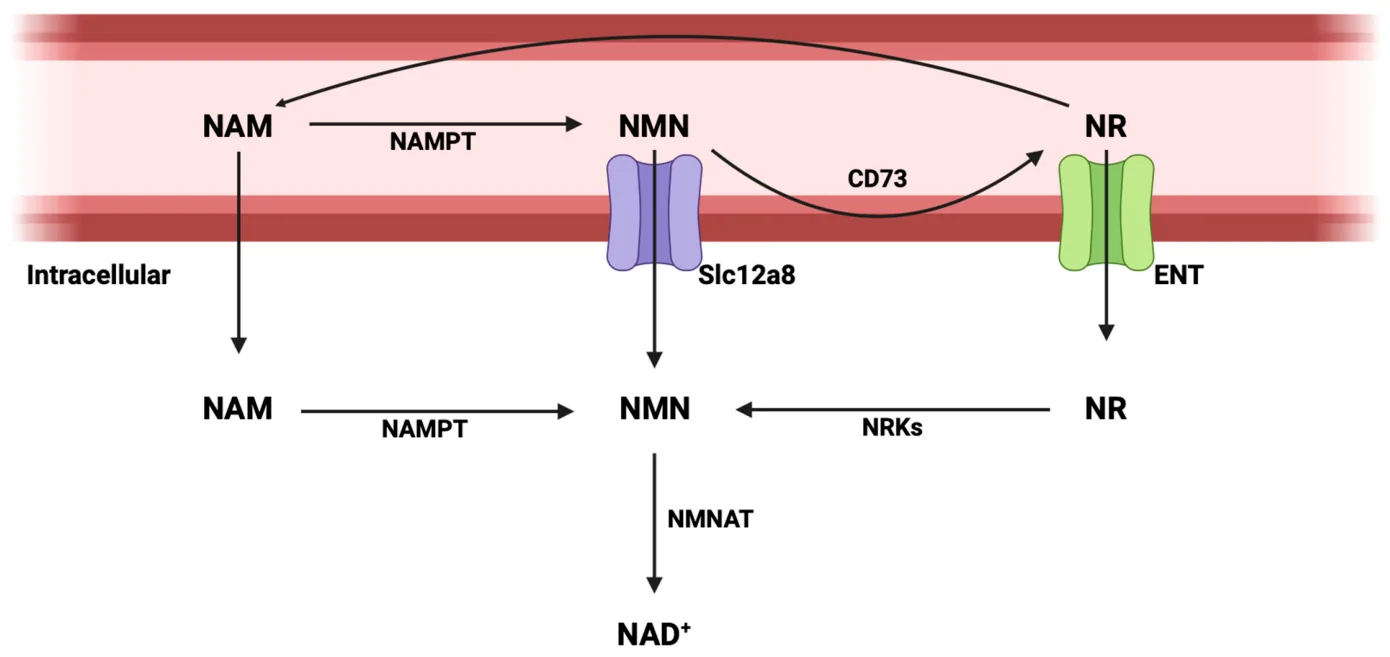
Cellular uptake of nicotinamide riboside, nicotinamide mononucleotide and nicotinamide. NMN can be transported into the cell through SLC12A8 or converted extracellularly to NR by CD73, while NR is transported through equilibrative nucleoside transporters (ENTs). Intracellularly, NAM and NR are converted to NMN, which is subsequently converted to NAD^+^. Arrows indicate the direction of metabolite transport or enzymatic conversion. CD73: ecto-5′-nucleotidase. ENT: equilibrative nucleoside transporters. NAM: nicotinamide. NAMPT: nicotinamide phosphoribosyltransferase. NAD^+^: nicotinamide adenine dinucleotide (oxidized). NMN: nicotinamide mononucleotide. NMNAT: nicotinamide mononucleotide adenylytransferases. NR: nicotinamide riboside. NRKs: nicotinamide riboside kinases. Slc12a8: solute carrier family 12 member 8.

Intracellular, NAD^+^ precursors enter NAD^+^ biosynthetic pathways, specifically the salvage pathway, to replenish intracellular NAD^+^ pools. The mechanisms by which these precursors enter the cell differ, as depicted in Figure 6. It has been proposed that NR can enter the cell through equilibrative nucleoside transporters (ENTs) and can also be converted to NAM in circulation or through hepatic first-pass metabolism [179,180]. NAM, due to its small and uncharged nature, can passively diffuse across the plasma membrane and subsequently enter the NAD^+^ salvage pathway [179,180]. In contrast, nicotinamide mononucleotide (NMN) uptake appears to be more complex due to its large molecule size. Slc12a8 has been identified as an NMN transporter to facilitate NMN uptake [181,182]. Additionally, NMN may undergo extracellular dephosphorylation to nicotinamide riboside (NR) by ectonucleotides such as CD73, allowing subsequent cellular uptake via nucleoside transporters [177].

#### 6.3.1. Nicotinamide Mononucleotide (NMN)

NMN undergoes intracellular metabolism to NAD^+^ by NMNATs. Supplementation with NMN increases NAD^+^ levels and improves metabolic function across multiple tissues. In mice, dietary administration of NMN (400 mg/kg/day for one week) significantly increased hepatic NAD^+^ levels [138]. Similarly, intraperitoneal administration of NMN (~500 mg/kg/day for 7–10 days) restored NAD^+^ pools depleted by HFD feeding, resulting in improved hepatic insulin sensitivity and glucose tolerance [137]. Long-term NMN supplementation (100–300 mg/kg/day in drinking water for 12 months) further demonstrated sustained increases in NAD^+^ levels, along with improvements in insulin sensitivity, plasma lipid profiles and whole-body energy metabolism without adverse effects [183].

NMN has also been shown to improve pancreatic β-cell function. A singular bolus of NMN (500 mg/kg, IP) enhanced GSIS and improved glucose tolerance in aged wildtype and β-cell-specific SIRT1 overexpression mice [184]. In mice fed a high-fructose diet, NMN treatment improved β-cell function [185]. Notably, knockdown of SIRT3 in INS-1 cells impaired β-cell function and abolished the protective effects of NMN, suggesting that NMN-mediated improvements are dependent, at least in part, on SIRT3 activity [128]. Maternal overnutrition and HFD feeding increases the risk of metabolic dysfunction characterized by glucose intolerance, increased adiposity and liver triglycerides in the offspring [186]. In offspring of obese, HFD-fed mothers, NMN treatment (500 mg/kg/day, IP) reduced adiposity and modestly improved glucose tolerance, suggesting a potential role in reversing developmental programming of metabolic disease [139].

In addition to metabolic benefits, NMN has demonstrated cardioprotective properties. In SIRT3-KO mice, NMN administration (500 mg/kg/day, IP for 14 days) increased myocardial NAD^+^ levels, enhanced SIRT1 expression, and improved cardiomyocyte and mitochondrial ultrastructure, ultimately ameliorating cardiac dysfunction via the SIRT1-PGC-1α pathway [187]. In a pressure overload model induced by TAC, cardiac NAD^+^ levels were reduced and associated with mitochondrial protein hyperacetylation [188]. NMN treatment (500 mg/kg once every three days, five days before surgery and four weeks post-surgery) restored NAD^+^ levels, reduced protein acetylation and attenuated cardiac hypertrophy [186].

Despite the promising preclinical studies, clinical studies have shown more modest effects. It is important to note that doses used in preclinical and clinical studies are reported according to the original studies and are not directly comparable across species because of differences in dosing strategies. In a randomized trial, oral administration of NMN (250 mg/day for 10 weeks) in postmenopausal women with prediabetes who were obese/overweight increased plasma NAD^+^ levels and improved muscle insulin sensitivity, but had no effect on blood glucose levels [189]. Similarly, 12-week supplementation with NMN (125 mg/day) in healthy individuals increased NAD^+^ metabolite levels in the serum and modestly improved arterial stiffness compared to placebo [190].

Overall, NMN supplementation consistently increases NAD^+^ levels and improves metabolic function in preclinical models across multiple tissues; however, evidence from human studies remains limited.

#### 6.3.2. Nicotinamide Riboside (NR)

NR is another NAD^+^ precursor that undergoes intracellular conversion to NMN by nicotinamide riboside kinases (NRKs) and subsequently to NAD^+^ by NMNATs (Figure 6) [138,191]. Supplementation with NR has been widely studied as a strategy to increase intracellular NAD^+^ levels and enhance sirtuin activity. Canto et al. demonstrated that dietary supplementation with NR (400 mg/kg/day for one week) significantly increased NAD^+^ levels in both intracellular and mitochondrial pools in the liver and skeletal muscle [138], which was associated with enhanced activity of SIRT1 and SIRT3 in vitro and in vivo, assessed by deacetylation of known target proteins [138]. Importantly, NR-mediated deacetylation was abolished by SIRT1 and SIRT3 knockdown in vitro, despite preserved NAD^+^ accumulation, indicating that these effects are mediated through sirtuin-dependent mechanisms. Furthermore, NR has demonstrated beneficial effects on whole-body metabolism. In male C57Bl/6J mice, NR supplementation (400 mg/kg/day for 12 weeks) reduced body weight and fat mass under HFD, without altering food intake and improved insulin-stimulated glucose disposal [138]. HFD-fed NR supplemented mice also showed higher mitochondrial cristae content; however, mitochondrial respiration was not measured [138]. Similarly, in mice fed a HFHS diet, NR supplementation (400 mg/kg/day) for 18 weeks (preventative group) or nine weeks (therapeutic group) reduced fat mass, improved glucose tolerance and insulin sensitivity and attenuated hepatic steatosis in both groups [192]. These effects were associated with improved mitochondrial function and increased expression of genes involved in β-oxidation, and were blunted in liver-specific SIRT1-KO suggesting an important role for SIRT1 in mediating these effects [192].

NR also shows protective effects in models of diabetic complications and liver injury. In a model of HFD and low-dose STZ induced diabetes, NR supplementation (3 g of NR chloride per kg of HFD food) improved glucose tolerance, reduced hepatic steatosis and protected against diabetic peripheral neuropathy [193]. In models of liver regeneration following partial hepatectomy, short-term NR supplementation (~500 mg/kg/day in drinking water for 14 days) increased hepatic NAD^+^ levels, reduced hepatic steatosis and promoted liver regeneration [194,195]. However, in this study, the loss of liver-specific SIRT1 or SIRT3 did not abolish the beneficial effects of NR, suggesting that NR may exert both sirtuin dependent and independent effects [194,195].

NR also shows promising effects on cardiac function and mitochondrial metabolism. Cardiomyocyte-specific deletion of *Nampt*, which reduces myocardial NAD^+^ levels, results in cardiac hypertrophy, and NR supplementation (500 mg/kg via oral gavage for four weeks) reversed these effects [196]. In a mouse model of HFpEF characterized by reduced NAD^+^ levels, impaired mitochondrial respiration and increased protein acetylation, NR supplementation (400 mg/kg/day for eight weeks) restored NAD^+^ levels, improved mitochondrial function and reversed the HFpEF phenotype [42]. Additionally, short-term NR supplementation (1.6 mg/mL in drinking water) during gestation increased mitochondrial complex IV, SIRT3 activity and reduced protein acetylation in fetal hearts exposed to hypoxia, although NAD^+^ levels were not increased in fetal cardiac tissue [197].

Despite these promising findings, the metabolic benefits of NR have not been consistently observed across studies. Differences in genetic background between C57Bl/6J and C57Bl/6N mouse strains may influence responses to NAD^+^ augmentation. C57Bl/6J mice have a spontaneous loss-of-function mutation in nicotinamide nucleotide transhydrogenase (*Nnt*), resulting in altered mitochondrial redox balance [198]. In a comparative study, Cartwright et al. demonstrated that NR supplementation failed to improve metabolic outcomes despite clear differences in baseline metabolic phenotypes in response to HFD feeding, highlighting variability in responses to NR [199]. This variability warrants further investigation into the response to NR in other diabetic and obesogenic mouse models such as *db*/*db* and *ob*/*ob.*

Clinical evidence also suggests potential cardiovascular benefits of NR supplementation. In a randomized, double-blind clinical trial of 90 individuals with peripheral artery disease, NR supplementation (1000 mg/day for six months) improved six-minute walk distance by 17.6 m compared with placebo [200]. However, this study used a prespecified one-sided significance threshold and included a relatively small cohort, highlighting the need for larger clinical trials to confirm these findings. Together with the variable metabolic responses observed in preclinical models, these findings suggest that the benefits of NR may depend on the underlying disease and metabolic context.

## 7. Future Directions

Despite significant advances in understanding sirtuin biology and NAD^+^ metabolism in cardiometabolic diseases, several critical gaps remain. One major limitation is the incomplete understanding of the functional relevance of lysine acetylation sites. Although proteomic studies have identified widespread acetylation across mitochondrial proteins [38,63,114,121], it remains unclear which specific sites are functionally important. In particular, the stoichiometry of acetylation required to influence enzyme activity is not well defined. Importantly, many studies lack site-specific mutagenesis approaches (lysine to glutamine amino acid substitutions) to directly assess the functional consequences of individual acetylation sites, making it difficult to establish causality between acetylation and changes in enzymatic activity and their impact on overall mitochondrial metabolism. For some enzymes, the functional consequences of specific acetylation sites have been investigated. For example, LCAD has a well-defined acetylation site, K42; however, this site is not located within the catalytic core of the enzyme [38]. Subsequently, K318 and K322 were identified as functionally relevant sites of acetylation for LCAD activity [39]. These sites are located adjacent to conserved amino acids involved in coordinating the FAD cofactor within the active site, suggesting that acetylation induces conformational changes impairing enzyme activity [39].

It also remains unclear whether acetylation occurs prior to mitochondrial import or within the mitochondrial matrix and to what extent this process is enzymatic versus non-enzymatic. In chronic overnutrition states, levels of acetyl-CoA are high in the mitochondria, driving increased acetylation in combination with reduced deacetylase activity. In the nucleus, increased activities of KATs may play a greater role [36]. Furthermore, it is not fully established whether mitochondrial protein hyperacetylation is a driver of metabolic dysfunction or develops secondary to mitochondrial stress during nutrient excess. Increased acetyl-CoA availability, altered NAD^+^ metabolism and reduced sirtuin activity promote mitochondrial protein hyperacetylation which subsequently impairs the activity of metabolic enzymes [39]. Conversely, mitochondrial dysfunction and altered substrate metabolism may themselves modify acetyl-CoA and NAD^+^ availability, thereby promoting further changes in protein acetylation. Establishing the temporal relationship between these events is important for determining whether mitochondrial hyperacetylation represents a causal mechanism of metabolic dysfunction or a consequence of altered mitochondrial metabolism.

It is important to note that sirtuin-mediated deacetylation does not operate in isolation; rather its physiological efficacy is linked to upstream mitonuclear balance, tissue-specific transcriptomes and cellular cofactor availability. Closely related to this is the limited understanding of NAD^+^ compartmentalization. The regulation and measurement of NAD^+^ pools across subcellular compartments, including the cytosol, nucleus and mitochondria remain technically challenging [142]. The extent to which these pools are functionally relevant and exert the most benefits due to NAD^+^ boosting is unclear. It is also unclear whether NR and NMN exert protective roles through SIRT1 and/or SIRT3. Finally, although NR and NMN have shown promising results in preclinical obesity, T2D and CVD, their translation to humans has been less consistent. A critical appraisal of human NR supplementation trials concluded that, despite increases in circulating NAD^+^ and related metabolites, few clinically relevant metabolic benefits have been consistently demonstrated [201]. However, many clinical studies have involved relatively small cohorts, short intervention periods or relatively healthy populations, highlighting the need for larger and longer-term trials in populations with established cardiometabolic disease. Moreover, it is unclear whether NAD^+^ precursors should be used as standalone therapies or as adjuncts to established interventions, such as lifestyle modification and first-line diabetes treatment metformin. Beyond identifying effective therapeutic targets, improving the delivery of sirtuin-modulating compounds represents another important translational consideration, particularly for compounds with limited bioavailability. Extracellular vesicles (EVs) are increasingly being investigated as drug-delivery vehicles in cardiovascular disease and may provide a strategy for improving the targeted delivery of therapeutic agents [202]. Whether EV-based approaches can improve the delivery and efficacy of sirtuin-targeting therapeutics remains to be determined.

Biological sex represents another important variable that remains insufficiently characterized in sirtuin research. Emerging evidence suggests that sirtuin expression and metabolic responses to sirtuin deficiency may differ between males and females. In SIRT3-KO mice, the hepatic response to HFD was sex-dependent, with males exhibiting SIRT3-dependent alterations across a broader range of metabolic parameters, whereas females showed greater effects on mitochondrial respiration and oxidative damage [203]. Future studies should therefore incorporate both sexes and determine whether sex influences sirtuin regulation and responses to sirtuin-targeting or NAD^+^-augmenting interventions.

Addressing these critical gaps is essential to fully elucidate the role of sirtuin-mediated acetylation in metabolic regulation. This remains a highly promising area of research with strong potential to uncover novel mechanisms and therapeutic targets for cardiometabolic disease.

## 8. Conclusions

Sirtuins have emerged as key regulators of cellular metabolism, linking nutrient availability to the control of protein acetylation and mitochondrial function. As NAD^+^-dependent deacetylases, their activity is intrinsically coupled to cellular energy and redox state. In cardiometabolic diseases, including obesity, T2D, CVD, and MASLD, dysregulation of sirtuin activity, particularly of SIRT1 and SIRT3, is closely associated with impaired metabolic flexibility and mitochondrial dysfunction. Future conceptual frameworks must increasingly bridge the gap between systemic cofactor economics and organ-specific metabolic priorities to fully decode the pleiotropic effects of sirtuin modulation. A central mechanism underlying these effects is the decline in NAD^+^ availability during nutrient excess, which limits sirtuin activity and promotes mitochondrial protein hyperacetylation. While pharmacological strategies aimed at directly activating sirtuins have shown promise, their mechanisms of action remain controversial. NAD^+^-augmenting approaches have demonstrated more consistent metabolic benefits in preclinical models, highlighting the importance of restoring NAD^+^ homeostasis to improve metabolic function. Collectively, these findings position sirtuins and NAD^+^ metabolism as critical regulators of energy metabolism and key targets for therapeutic intervention in cardiometabolic disease.

## Figures and Tables

**Table 1:** Non-mitochondrial sirtuin activity and localization.

Sirtuin	Activity	Location
SIRT1	Deacetylation	Nucleus and cytosol
SIRT2	Deacetylation	Cytosol
SIRT6	Mono-ADP-ribosylation, deacetylation	Nucleus
SIRT7	Deacetylation	Nucleolar

SIRT1: sirtuin 1. SIRT2: sirtuin 2. SIRT6: sirtuin 6. SIRT7: sirtuin 7.

**Table 2:** Mitochondrial sirtuin activity and localization.

Sirtuin	Activity	Location
SIRT3	Deacetylation	Mitochondria
SIRT4	Mono-ADP-ribosylation, weak deacetylation	Mitochondria
SIRT5	Desuccinylation, demalonylation, deglutarylation, weak deacetylation	Mitochondria

SIRT3: sirtuin 3. SIRT4: sirtuin 4. SIRT5: sirtuin 5.

**Table 3:** Roles of SIRT1 and SIRT3 in experimental animal models of cardiovascular disease.

Sirtuin	Cardiovascular Condition	Experimental Model	Major Findings	Ref.
SIRT1	Diabetic cardiomyopathy	Cardiac-specific SIRT1-KO	SIRT1 deletion promotes cardiac hypertrophy, impairs cardiac function and insulin resistance	[77]
SIRT1	Diabetic cardiomyopathy	*db*/*db* mice and recombinant SIRT1 overexpression	Reduced cardiac SIRT1 and impairs cardiac function; restoration of SIRT1 improves cardiac function	[79]
SIRT1	Ischemia/reperfusion injury	Cardiac-specific SIRT1 deletion and overexpression; I/R injury	SIRT1 deletion increases infarct size, whereas SIRT1 overexpression protectives against I/R injury	[80]
SIRT1	Cardiac hypertrophy	Neonatal rat cardiomyocytes	SIRT1 overexpression attenuates phenylephrine-induced hypertrophic markers	[81]
SIRT1	Cardiomyopathy	High- and moderate-level cardiac-specific SIRT1 overexpression mice	High expression of SIRT1 causes dilated cardiomyopathy and mitochondrial dysfunction whereas moderate level protects against hypertrophy	[82,83]
SIRT3	Cardiac hypertrophy	Whole-body SIRT3-KO mice	SIRT3 deficiency promotes cardiac hypertrophy, fibrosis and mitochondrial dysfunction	[84,85]
SIRT3	Ischemia/reperfusion injury	SIRT3-KO mice and I/R injury	SIRT3 deficiency impairs mitochondrial respiration, reduces functional recovery and increases infarct size following I/R	[86]
SIRT3	Myocardial infarction	SIRT3-KO mice and MI model	SIRT3 deficiency exacerbates post-MI cardiac dysfunction, whereas increased SIRT3 expression improves cardiac recovery	[87]
SIRT3	Cardiac hypertrophy	TAC; SIRT3-KO	SIRT3 deficiency impairs FAO and promotes myocardial lipid accumulation and cardiac dysfunction	[88]
SIRT3	Doxorubicin-induced cardiomyopathy	H9c2 cells and cardiac-specific SIRT3 Tg mice	SIRT3 overexpression reduces oxidative stress in cells and Tg mice and improves mitochondrial respiratory function and reduces hyperacetylation	[89,90]
SIRT3	Cardiometabolic dysfunction	HFD and SIRT3-KO	SIRT3 deficiency increased acetylation of FAO enzymes and altered cardiac substrate metabolism	[76]
SIRT3	HFpEF	Cardiac-specific SIRT3-KO mice	SIRT3 deletion accelerates diastolic dysfunction	[42]

FAO: fatty acid oxidation. HFD: high-fat diet. HFpEF: heart failure with preserved ejection fraction. I/R: ischemia/reperfusion. KO: knock-out. SIRT1: sirtuin 1. SIRT3: sirtuin 3. TAC: transverse aortic constriction. Tg: transgenic.

**Table 4:** Summary of SIRT1 and SIRT3 in animal models of metabolic disease.

Sirtuin	Metabolic Disease	Experimental Model	Major Findings	Ref.
SIRT1	Obesity and insulin resistance	Global SIRT1 transgenic mice, HFD	SIRT1 overexpression improves glucose tolerance and insulin sensitivity and reduces hepatic glucose production during HFD feeding	[111]
SIRT1	Hepatic metabolic dysfunction	Liver-specific SIRT1-KO mice	SIRT1 deletion results in hyperglycemia, increased hepatic glucose production and impaired peripheral insulin signaling	[96]
SIRT1	Obesity and insulin resistance	Adipocyte-specific SIRT1-KO mice, HFD	SIRT1 deletion results hyperacetylation, glucose intolerance, hyperinsulinemia and insulin resistance	[92]
SIRT1	Skeletal muscle metabolic dysfunction	Skeletal muscle-specific SIRT1-KO mice	SIRT1 deletion impairs mitochondrial respiration and reduces mitochondrial content	[103]
SIRT1	β-cell dysfunction	β-cell-specific SIRT1-KO mice	SIRT1 deletion impairs glucose-stimulated insulin secretion	[94]
SIRT3	Obesity and insulin resistance	Whole-body SIRT3-KO mice, HFD	SIRT3 deficiency results in mitochondrial protein hyperacetylation with reduced mitochondrial function and phenocopies obesity, insulin resistance	[40,72,114]
SIRT3	Hepatic metabolic dysfunction	Liver-specific SIRT3-KO mice	SIRT3 deletion causes marked mitochondrial protein hyperacetylation but mild metabolic abnormalities	[121]
SIRT3	Adipose metabolic dysfunction	Adipocyte-specific SIRT3-KO mice, HFD	No significant metabolic abnormalities	[124]
SIRT3	Skeletal muscle metabolic dysfunction	Skeletal muscle-specific SIRT3-KO mice	SIRT3 deletion increased mitochondrial protein acetylation without metabolic abnormalities	[121]

HFD: high-fat diet. KO: knock-out. SIRT1: sirtuin 1. SIRT3: sirtuin 3.

**Table 5:** Preclinical and clinical development of pharmacological sirtuin modulators.

Compound	Target	Proposed Mechanism of Sirtuin Modulation	Development Stage	Major Findings	Limitations	Ref.
Resveratrol	SIRT1	Indirect metabolic modulator	Preclinical & clinical	Improved mitochondrial function, glucose homeostasis and cardiometabolic outcomes in several preclinical models.	Low bioavailability, pleiotropic effects and controversy regarding direct SIRT1 activation	[144,145,146,147,148]
Quercetin	SIRT1	Reported SIRT1 activation in vitro	Preclinical & clinical	Improved glucose homeostasis and β-cell function in preclinical models	Low bioavailability and uncertain specificity for SIRT1	[159,160]
Honokiol	SIRT3	Enhances SIRT3 expression/signaling	Preclinical	Improved mitochondrial function and reduced oxidative stress in cardiac and metabolic models	Pleiotropic effects and limited clinical validation; direct activation not established	[41,162]
SRT1720	SIRT1	Reported SIRT1-activating compound	Preclinical	Improved metabolic function and protected against HFD-induced metabolic dysfunction in rodents	Direct SIRT1 activation and target specificity remain controversial	[173]
SRT2104	SIRT1	Synthetic SIRT1-activating compound	Clinical (Phase II)	Well tolerated in T2D but did not improve glucose or insulin control; improved arterial stiffness in healthy smokers	Limited metabolic efficacy and variable pharmacokinetics; mechanism of direct activation remains debated	[169,170]
2-APQC	SIRT3	Direct allosteric SIRT3 activator	Preclinical	Allosteric activation of SIRT3 increased deacetylase activity and improved mitochondrial function	Limited in vivo validation; specificity and long-term safety remain unclear	[174]
SZC-6	SIRT3	Direct allosteric SIRT3 activator	Preclinical	SIRT3 activation improved mitochondrial function and reduced oxidative stress	Limited in vivo validation; specificity, pharmacokinetics and safety require further investigation	[175]

2-APQC: 2-acetyl-4-phenylquinoline. HFD: high-fat diet. SIRT1: sirtuin 1, SIRT3: sirtuin 3. SRT1720: synthetic SIRT1-activating compound. SRT2104: synthetic SIRT1-activating compound. T2D: type 2 diabetes.

## Data Availability

No new data were created or analyzed in this study. Data sharing is not applicable to this article.
